# In-hospital outcomes of SARS-CoV-2-infected health care workers in the COVID-19 pandemic first wave, Quebec, Canada

**DOI:** 10.1371/journal.pone.0272953

**Published:** 2022-08-24

**Authors:** Ilyse Darwish, Luke B. Harrison, Ana Maria Passos-Castilho, Annie-Claude Labbé, Sapha Barkati, Me-Linh Luong, Ling Yuan Kong, Marc-Antoine Tutt-Guérette, James Kierans, Cécile Rousseau, Andrea Benedetti, Laurent Azoulay, Christina Greenaway

**Affiliations:** 1 Division of Infectious Diseases, McGill University, Montréal, Quebec, Canada; 2 Centre for Clinical Epidemiology, Lady Davis Institute, Jewish General Hospital, Montréal, Quebec, Canada; 3 Department of Medicine & Division of Infectious Diseases, Hôpital Maisonneuve-Rosemont, CIUSSS de l’Est-de-l’Île-de-Montréal, Montréal, Quebec, Canada; 4 Department of Microbiology, Infectiology and Immunology, Université de Montréal, Montréal, Quebec, Canada; 5 Département des Laboratoires de Biologie Médicale, Grappe Optilab-CHUM, Centre Hospitalier de l’Université de Montréal (CHUM), Montreal, Canada; 6 Department of Medicine & Division of Infectious Diseases, McGill University Health Center (MUHC), McGill University, Montréal, Quebec, Canada; 7 Research Institute of the McGill University Health Centre, Montréal, Quebec, Canada; 8 Department of Medicine, McGill University, Montréal, Quebec, Canada; 9 Department of Medicine & Division of Infectious Diseases CHUM, Montréal, Quebec, Canada; 10 Department of Medicine & Division of Infectious Diseases, Jewish General Hospital, McGill University, Montréal, Quebec, Canada; 11 Department of Psychiatry, McGill University Health Center, McGill University, Montréal, Quebec, Canada; 12 SHERPA University Institute, CIUSSS du Centre-Ouest-de-l’Île-de-Montréal, Montréal, Quebec, Canada; 13 Department of Epidemiology, Biostatistics and Occupational Health, McGill University, Montréal, Quebec, Canada; University of Palermo, ITALY

## Abstract

**Background:**

Health care workers (HCW), particularly immigrants and ethnic minorities are at increased risk for SARS-CoV-2 infection. Outcomes during a COVID-19 associated hospitalization are not well described among HCW. We aimed to describe the characteristics of HCW admitted with COVID-19 including immigrant status and ethnicity and the associated risk factors for Intensive Care unit (ICU) admission and death.

**Methods:**

Adults with laboratory-confirmed community-acquired COVID-19 hospitalized from March 1 to June 30, 2020, at four tertiary-care hospitals in Montréal, Canada were included. Demographics, comorbidities, occupation, immigration status, country of birth, ethnicity, workplace exposures, and hospital outcomes (ICU admission and death) were obtained through a chart review and phone survey. A Fine and Gray competing risk proportional hazards model was used to estimate the risk of ICU admission among HCW stratified by immigrant status and region of birth.

**Results:**

Among 1104 included persons, 150 (14%) were HCW, with a phone survey participation rate of 68%. HCWs were younger (50 vs 64 years; *p*<0.001), more likely to be female (61% vs 41%; *p*<0.001), migrants (68% vs 55%; *p*<0.01), non-White (65% vs 41%; *p*<0.001) and healthier (mean Charlson Comorbidity Index of 0.3 vs 1.2; *p*<0.001) compared to non-HCW. They were as likely to be admitted to the ICU (28% vs 31%; *p* = 0.40) but were less likely to die (4% vs. 17%; *p*<0.001). Immigrant HCW accounted for 68% of all HCW cases and, compared to Canadian HCW, were more likely to be personal support workers (PSW) (54% vs. 33%, *p*<0.01), to be Black (58% vs 4%) and to work in a Residential Care Facility (RCF) (59% vs 33%; *p* = 0.05). Most HCW believed that they were exposed at work, 55% did not always have access to personal protective equipment (PPE) and 40% did not receive COVID-19-specific Infection Control (IPAC) training.

**Conclusion:**

Immigrant HCW were particularly exposed to COVID-19 infection in the first wave of the pandemic in Quebec. Despite being young and healthy, one third of all HCW required ICU admission, highlighting the importance of preventing workplace transmission through strong infection prevention and control measures, including high COVID-19 vaccination coverage.

## Introduction

The COVID-19 pandemic has disproportionately affected certain populations resulting in higher risk of infection, and associated morbidity and mortality. Health care workers (HCW), other essential workers, those living in economically deprived neighborhoods, ethnic minorities, and migrants have been the most affected [1–3]. HCW during the first wave of COVID-19 had increased exposure to SARS-CoV-2 due largely to direct contact with infected patients, lack of access to adequate personal protective equipment (PPE) and lack of COVID-19-tailored infection prevention and control (IPAC) training [2,4]. Among other groups of essential workers, increased SARS-CoV-2 exposure resulted from crowded living and working conditions, use of public transport, the inability to work from home due to the front-facing nature of their occupation, or the inability to take time off due to limited financial resources [5,6].

At the beginning of the pandemic HCW were more likely to be infected with SARS-CoV-2 and were 2–3 times as likely to be hospitalized for COVID-19 compared to the general population due to increased exposure [2,7,8]. The risk among HCW however, was not evenly distributed and COVID-19 associated in-hospital outcomes varied between studies [8,9]. Low paid personal support workers (PSW), particularly those working with elderly patients in Long Term Care Facilities (LTCF) in Canada, were at higher risk [10]. PSW have a 1.8 fold and 3.3 fold greater risk of SARS-CoV-2 infection compared to nurses and doctors, respectively [10]. Migrant HCW were also disproportionately affected by COVID-19 with Black, Asian and Hispanic HCW twice as likely to test positive for SARS-CoV-2 than their White colleagues in the UK and the US [11]. This may be due to the fact that migrants are more likely to be PSW or have other socio-economic disparities [12–15]. Once hospitalized, the likelihood of admission to the ICU and in-hospital mortality among HCW differed across studies and there is no data on these outcomes among migrant HCW [8,9]. This study aimed to address risk factor and outcome data gaps among HCW admitted with COVID-19. It had the two following goals: 1) to compare demographic including immigration status, country of origin and ethnicity, clinical characteristics, and outcomes between HCW and non-HCW hospitalized for COVID-19, 2) to compare demographic characteristics, outcomes, and workplace related risk factors for COVID-19 infection amongst Canadian-born HCW and immigrant HCW hospitalized for COVID-19. These results will identify which HCW are at greatest risk for COVID-19 and may inform infection control, public health and vaccination policy, and health resource allocation.

## Methods

### Cohort

We performed a retrospective cohort study of all patients ≥18 years of age hospitalized with laboratory-confirmed community acquired SARS-CoV-2 in four major tertiary care hospitals in Montréal, Canada. The participating hospitals serve a population of ~1.2 million people, of whom 32% are first-generation immigrants and accounted for half of the hospitalizations in Montreal during the study period [16,17]. Patients admitted from March 1 to June 30, 2020, during the first wave of the COVID-19 pandemic in Montréal were included and followed until August 31, 2020. HCW status and specific occupation were identified in the medical records and confirmed in a phone survey. Nasopharyngeal swabs or lower respiratory tract specimens were tested with a PCR test developed and validated by the Laboratoire de santé publique du Québec, targeting the SARS-CoV-2 envelope gene or a Health-Canada authorized commercial assay [18]. All nosocomial SARS-CoV-2 infections, defined as patients who developed SARS-CoV-2 infection seven or more days after admission, were excluded. Patients admitted from a Residential Care Facility for the elderly were also excluded.

### Data collection

Our analysis is based on two sources of information: 1) the electronic medical record to obtain data on demographic characteristics, HCW status, HCW occupation, and medical information including past medical history, date of onset of symptoms, severity of illness, immigrant status and country of origin and outcomes (ICU admission, intubation, and death); 2) a semi-structured phone survey, with interpreters when required, was conducted after hospital discharge with patients or a next of kin in the event of death. Data on self-identified ethnicity, SARS-CoV-2 exposure at home and work, access to PPE and IPAC training, and validation of HCW status and occupation was collected. HCW occupation was classified as personal support workers (PSW), auxiliary nurse, nurse, physician or medical student, other occupations with patient contact (respiratory therapy, pharmacy staff, social workers), and those without patient contact (administrative staff and managers, housekeeping and kitchen staff). A modified Charlson Comorbidity score, excluding diabetes, was estimated [19,20]. Diabetes was included as a separate variable given the increased prevalence of diabetes in certain ethnic groups and the association with poor COVID-19 outcomes [21,22]. For patients where the date of onset of symptoms was not available, the date of the first positive SARS-CoV-2 PCR test was used. We calculated the National Early Warning Score 2 (NEWS2) score based on admission vital signs to measure disease severity [23]. Neighborhood material and social deprivation scores were assigned using the Canadian Material and Social Deprivation Index (MSDI) based on the patients’ home address [24]. Immigrants were defined as persons born outside of Canada. Region of birth was grouped into the World Bank classification (East Asia/Pacific, South Asia, Middle East/North Africa, Sub-Saharan Africa, Europe/US, Latin America, Caribbean and Canada). Self-identified ethnicity was classified as White, Black, Asian, Latino, Middle East/North African, Mixed and Other. Missing ethnicity was imputed by region of birth data; White (Canada + Europe/US), Black (Caribbean + Sub-Saharan Africa), Asian (East Asia/Pacific + South Asia), Latino (Latin America), and Middle East/North African (Middle East/North Africa). This classification was 90% accurate based on the 60% self-reported ethnicity collected in the phone survey. The primary outcomes were admission to ICU and in-hospital mortality. Secondary outcomes were length of hospital stay, ICU length of stay, and need for mechanical ventilation. We classified Residential Care Facilities (RCF) for the elderly as either 1) CHSLDs (centre d’hébergement et de soins de longue durée) which are Long Term Care Facilities providing 24-hour registered nurse coverage and in which residents require more than three hours of nursing care per day, and 2) RPAs (résidence privées pour aînés) private facilities where older adults require a range of care from low (assistance with some activities of daily living), intermediate or high (requiring hours of nursing care) [25–27].

### Statistical analysis

Descriptive analyses were performed for patient characteristics and study outcomes, stratified by HCW vs. non-HCW. A similar descriptive analysis among only HCW was conducted and stratified by immigrant status, region of birth and ethnicity. Means are presented with standard deviation (SD) and medians with interquartile range (IQR). Statistical significance for comparisons was assessed by ANOVA or Kruskal–Wallis for continuous variables and chi-square tests or Fisher Exact test for categorical variables, with a significance level set at *p* < 0.05. Adjusted hazard ratios (aHR) of ICU admission among HCW by immigrant status, ethnicity, and region of birth and adjusted for age, sex, modified Charlson comorbidity score, diabetes, and MSDI, with calendar month as strata, were estimated with a Fine and Gray competing risk proportional hazards models [28]. In-hospital death was considered a competing risk for ICU admission. Follow-up time began with the date of admission to hospital and ended at the date of in-hospital death, date of hospital discharge or the end of the study observation (August 31, 2020). The proportional hazard assumption was verified with log-log survival curves and Schoenfeld residuals for all variables included in the models. There was no violation of the proportional hazards assumption. All analyses were performed with SAS software version 9.4 (SAS Institute Inc.; Cary, NC).

### Ethics approval

This study was submitted to and approved by the following Research Ethics Boards: CIUSSS de l’Est-de-l’Île-de-Montréal, CIUSSS West-Central Montréal, McGill University Health Centre, and Centre Hospitalier de l’Université de Montréal. Verbal consent was obtained by telephone for all survey participants or their next of kin in the event of death. This process was approved by each ethics board to reduce the risk of COVID-19 transmission. A copy of the consent form was provided to each participant.

## Results

### Healthcare workers and non-healthcare workers

A total of 1104 patients with a laboratory-confirmed SARS-CoV-2 infection admitted between March 1, 2020, and June 30, 2020, were included in the study. Among these patients, 150 (13.6%) were HCW (Table 1). HCW were significantly younger (mean 49.6 vs 63.9 years; p*<*0.001), more likely to be female (60.7% vs 41.4%; p<0.001), to have a lower mean Charlson Comorbidity Index (mean 0.3 vs. 1.2; p<0.001), had a similar deprivation index (mean 3.9 vs 3.9; *p* = 0.92) and NEWS2 disease severity index (mean 4.4 vs. 4.4; *p* = 0.88) on admission compared to non-HCW. HCW were more likely to be immigrants (68.9% vs 55.0%; *p*<0.01) and non-White (64.6% vs 40.9%; *p*<0.001). Most HCW identified as Black (41.0%) and White (35.4%), followed by Asian (11.1%), Middle Eastern/North African (7.6%), and Latino (4.9%). HCW were primarily PSW (47.6%) and nurses (15.9%).

**Table 1:** Chart review data stratified by healthcare worker vs. non–healthcare worker.

	*Overall N(%)*	*Health Care Worker N(%)*	*Non-Health Care Worker N(%)*	*p-value*
**Total**	1104 (100)	150 (13.6)	954 (86.4)	
**CHARACTERISTICS ON ADMISSION**				
**Age (years)**				<0.001
Mean (SD)	62.0 (17.5)	49.6 (11.4)	63.9 (17.5)	
**Sex**				<0.001
Male	618 (56.0)	59 (39.3)	559 (58.6)	
Female	486 (44.0)	91 (60.7)	395 (41.4)	
**Type of HCW (N = 145)**				
Personal Support Worker		69 (47.6)		
Nurse		23 (15.9)		
Auxiliary nurse		10 (6.9)		
Physician/Medical Student		8 (5.5)		
Other Patient Contact^1^		12 (8.3)		
Other Non-Patient Contact^2^		23 (15.9)		
**Immigrant Status (N = 1093)**				<0.01
Canadian-born	471 (43.1)	46 (31.1)	425 (45.0)	
Immigrant	622 (56.9)	102 (68.9)	520 (55.0)	
**Region of Birth (N = 1052)**				<0.001
Canada	471 (44.8)	46 (31.9)	425 (46.8)	
East Asia and Pacific	53 (5.0)	12 (8.3)	41 (4.5)	
South Asia	39 (3.7)	2 (1.4)	37 (4.1)	
Middle East and North Africa	80 (7.6)	11 (7.6)	69 (7.6)	
Sub Saharan Africa	63 (6.0)	20 (13.9)	43 (4.7)	
Europe and United-States	146 (13.9)	9 (6.3)	137 (15.1)	
Latin America	45 (4.3)	6 (4.2)	39 (4.3)	
Caribbean	155 (14.7)	38 (26.4)	117 (12.9)	
**Ethnicity (N = 1054)**				<0.001
White	589 (55.9)	51 (35.4)	538 (59.1)	
Black	219 (20.8)	59 (41.0)	160 (17.6)	
Latino	52 (4.9)	7 (4.9)	45 (4.9)	
Middle Eastern North African	81 (7.7)	11 (7.6)	70 (7.7)	
Asian	99 (9.4)	16 (11.1)	83 (9.1)	
Other/Mixed	14 (1.3)		14 (1.5)	
**Language Barrier**				<0.001
Yes	101 (9.1)		101 (10.6)	
No	1003 (90.9)	150 (100.0)	853 (89.4)	
**Material and Social Deprivation Index (N = 1040)**				0.92
Mean (SD)	3.9 (1.3)	3.9 (1.3)	3.9 (1.3)	
**Charlson Comorbidity Index** ^**3**^				<0.001
Mean (SD)	1.1 (1.7)	0.3 (0.8)	1.2 (1.8)	
**Comorbidities**				
Hypertension	565 (51.2)	55 (36.7)	510 (53.5)	<0.001
Coronary Artery Disease	163 (14.8)	7 (4.7)	156 (16.4)	<0.001
Asthma	112 (10.1)	23 (15.3)	89 (9.3)	0.02
Chronic Pulmonary Disease	130 (11.8)	8 (5.3)	122 (12.8)	<0.01
Diabetes	341 (30.9)	33 (22.0)	308 (32.3)	0.01
Immunosuppression	88 (8.0)	6 (4.0)	82 (8.6)	0.07
**Length of Symptoms (days) (N = 1057)**				<0.001
Mean (SD)	7.1 (5.4)	9.1 (5.5)	6.8 (5.3)	
**NEWS2**				0.88
Mean (SD)	4.4 (3.2)	4.4 (3.2)	4.4 (3.2)	
**OUTCOMES**				
**ICU Admission**	342 (31.0)	42 (28.0)	300 (31.4)	0.40
**Length of ICU Stay (days)**				0.39
Median (IQR)	10.0 (5.0–20.0)	8.0 (5.0–18.0)	11.0 (5.0–21.0)	
**Intubated**	219 (64.0)	22 (52.4)	197 (65.7)	0.09
**Length of Intubation (days) (N = 219)**				0.76
Median (IQR)	13.0 (8.0–22.0)	14.5 (6.0–19.0)	13.0 (8.0–22.0)	
**Length of Hospital Stay (days)**				<0.001
Median (IQR)	10.0 (5.0–20.0)	7.0 (5.0–12.0)	11.0 (6.0–22.0)	
**Disposition at Discharge**				<0.001
Death	167 (15.1)	6 (4.0)	161 (16.9)	
Discharged Home	919 (83.2)	144 (96.0)	775 (81.2)	
Still Admitted	18 (1.6)		18 (1.9)	

Abbreviations: IQR, interquartile range. NEWS2, National Early Warning Score 2. SD, standard deviation.

^1^Other Patient contact: pharmacy staff (2), social worker (2), alternative medicine (1), counsellor (1), dentist (1), neuronavigator (1), nursing student (1), respiratory therapist (1), RPA worker (1), transportation (1).

^2^Other Non–patient contact: administrative staff (9), manager (4), kitchen staff (3), housekeeping attendant (2), security (2), chemist (1), sterilization (1), recreative technician (1).

^3^Does not include diabetes.

### Health care workers by immigration status and ethnicity

A total of 68.0% (102/150) of all HCW were immigrants. Immigrant and non-immigrant HCW had a similar age (mean, 50.2 vs 48.5; *p* = 0.41), sex (64.7% vs 50.0% females; *p* = 0.09), mean Charlson Comorbidity Index (0.3 vs 0.3; *p* = 0.89), and deprivation index (4.0 vs 3.6; *p* = 0.11) (Table 2). Immigrant HCW were more likely to be PSW (54.0% vs 32.6%; *p*<0.01) compared to their Canadian-born colleagues. Ethnicity was significantly different between immigrants and Canadian-born HCW (*p*<0.001). Immigrant HCW were more likely to be Black (58.2% vs 4.3%; *p*<0.001) whereas Canadian HCW were more likely to be White (87.0% vs. 11.2%; *p*<0.001). Most immigrant HCW were born in the Caribbean (38.8%), Sub-Saharan Africa (20.4%), East Asia/Pacific (12.2%) or the Middle East/North Africa (11.2%).

**Table 2:** Health care worker chart review data stratified by immigration status.

	*Overall N(%)*	*Canadian- Born N(%)*	*Immigrant N(%)*	*p-value*
**Total**	150 (100)	46 (30.7)	102 (68.0)	
**CHARACTERISTICS ON ADMISSION**				
**Age (years)**				0.41
Mean (SD)	49.6 (11.4)	48.5 (12.1)	50.2 (10.9)	
**Sex**				0.09
Male	59 (39.3)	23 (50.0)	36 (35.3)	
Female	91 (60.7)	23 (50.0)	66 (64.7)	
**Type of HCW (N = 145)**				<0.01
Personal Support Worker	69 (47.6)	14 (32.6)	54 (54.0)	
Nurse	23 (15.9)	5 (11.6)	18 (18.0)	
Auxiliary nurse	10 (6.9)	2 (4.7)	8 (8.0)	
Physician/Medical Student	8 (5.5)	3 (7.0)	5 (5.0)	
Other Patient Contact^1^	12 (8.3)	10 (23.3)	2 (2.0)	
Other Non-Patient Contact^2^	23 (15.9)	9 (20.9)	13 (13.0)	
**Region of Birth (N = 146)**				
Canada		46 (100)		
East Asia and Pacific			12 (12.2)	
South Asia			2 (2.0)	
Middle East and North Africa			11 (11.2)	
Sub Saharan Africa			20 (20.4)	
Europe and United-States			9 (9.2)	
Latin America			6 (6.1)	
Caribbean			38 (38.8)	
**Ethnicity (N = 144)**				<0.001
White	51 (35.4)	40 (87.0)	11 (11.2)	
Black	59 (41.0)	2 (4.3)	57 (58.2)	
Latino	7 (4.9)	2 (4.3)	5 (5.1)	
Middle Eastern/North African	11 (7.6)		11 (11.2)	
Asian	16 (11.1)	2 (4.3)	14 (14.3)	
**Material and Social Deprivation Index (N = 143)**				0.11
Mean (SD)	3.9 (1.3)	3.6 (1.4)	4.0 (1.2)	
**Charlson Comorbidity Index** ^**3**^				0.89
Mean (SD)	0.3 (0.8)	0.3 (0.8)	0.3 (0.8)	
**Comorbidities**				
Hypertension	55 (36.7)	17 (37.0)	38 (37.3)	0.97
Coronary Artery Disease	7 (4.7)	3 (6.5)	4 (3.9)	0.68
Asthma	23 (15.3)	9 (19.6)	14 (13.7)	0.36
Chronic Pulmonary Disease	8 (5.3)	3 (6.5)	5 (4.9)	0.70
Diabetes	33 (22.0)	12 (26.1)	21 (20.6)	0.46
Immunosuppression	6 (4.0)	2 (4.3)	4 (3.9)	1.00
**Length of Symptoms (days) (N = 149)**				0.87
Mean (SD)	9.1 (5.5)	9.1 (6.0)	9.2 (5.3)	
**NEWS2**				0.23
Mean (SD)	4.4 (3.2)	3.9 (2.8)	4.6 (3.4)	
**OUTCOMES**				
**ICU Admission**	42 (28.0)	9 (19.6)	33 (32.4)	0.11
**Length of ICU Stay (days)**				0.78
Median (IQR)	8.0 (5.0–18.0)	7.0 (6.0–10.0)	9.0 (5.0–20.0)	
**Intubated**	22 (52.4)	6 (66.7)	16 (48.5)	0.33
**Length of Intubation (days) (N = 22)**				0.20
Median (IQR)	14.5 (6.0–19.0)	10.0 (4.0–15.0)	15.0 (7.0–32.5)	
**Length of Hospital Stay (days)**				0.89
Median (IQR)	7.0 (5.0–12.0)	7.5 (6.0–10.0)	7.0 (4.0–13.0)	
**Disposition at Discharge**				0.67
Death	6 (4.0)	1 (2.2)	5 (4.9)	
Discharged Home	144 (96.0)	45 (97.8)	97 (95.1)	

Abbreviations: IQR, interquartile range. NEWS2, National Early Warning Score 2. SD, standard deviation.

^1^Other Patient contact: pharmacy staff (2), social worker (2), alternative medicine (1), counsellor (1), dentist (1), neuronavigator (1), nursing student (1), respiratory therapist (1), RPA worker (1), transportation (1).

^2^Other Non–patient contact: administrative staff (9), manager (4), kitchen staff (3), housekeeping attendant (2), security (2), chemist (1), sterilization (1), recreative technician (1).

^3^Does not include diabetes.

2 of 150 (1.3%) patients missing immigration status not shown: mean age of 43.0 years (SD 22.6), 100% female, 0% admitted to ICU, 0% died.

### Intensive care unit admission and in-hospital mortality

HCW were as likely as non-HCW to be admitted to the ICU (28.0% vs 31.4%; *p* = 0.40) (Table 1). Overall, immigrant HCW were not at increased risk of admission to ICU compared to Canadian-born HCW after adjusting for age, sex, medical comorbidities and deprivation index (aHR 1.69, 0.81–3.54). However, in the model stratified by region of birth shown in Table 3, immigrants from East Asia and the Pacific were at higher risk of ICU admission compared to Canadian-born HCW (aHR 3.59, 1.19–10.86). HCW were less likely to die compared to non-HCW (4.0% vs 16.9%; *p*<0.001). A total of six HCW died, of whom five were immigrants.

**Table 3:** Hazards of admission to the Intensive Care Unit among health care workers hospitalized with COVID–19 by immigrant status, region of birth and ethnicity.

			*Immigration Status*		*Region of Birth*		*Ethnicity*	
*Variable*	*No*. *of patients*	*No*. *(% total) of patients admitted to ICU*	*HR* *(95% CI)*	*aHR* *(95% CI)*	*HR* *(95% CI)*	*aHR* *(95% CI)*	*HR* *(95% CI)*	*aHR* *(95% CI)*
**Immigrant status (Ref: Canadian)**	46	9 (19.6)	1.0	1.0				
Non Canadian	102	33 (32.4)	1.81 (0.87–3.80)	1.69 (0.81–3.54)				
**Region of Birth (Ref: Canada)**	46	9 (19.6)			1.0	1.0	1.0	1.0
Caribbean	38	11 (28.9)			1.50 (0.62–3.61)	1.32 (0.52–3.34)		
East Asia and Pacific	12	5 (41.7)			2.61 (0.87–7.84)	3.59 (1.19–10.86)		
Europe and United-States	9	3 (33.3)			1.73 (0.47–6.44)	2.07 (0.53–8.06)		
Latin America	6	3 (50.0)			3.02 (0.81–11.18)	3.16 (0.80–12.46)		
Middle East and North Africa	11	2 (18.2)			1.12 (0.24–5.20)	0.51 (0.06–4.02)		
South Asia	2	0 (0.0)			0.00 (0.00–0.00)	0.00 (0.00–0.00)		
Sub Saharan Africa	20	8 (40.0)			2.47 (0.95–6.41)	2.17 (0.80–5.90)		
**Ethnicity (Ref: White)**	51	10 (19.6)			1.0	1.0	1.0	1.0
Asian	16	6 (37.5)					2.14 (0.77–5.90)	1.98 (0.73–5.39)
Black	59	20 (33.9)					1.85 (0.87–3.96)	1.45 (0.64–3.28)
Latino	7	3 (42.9)					2.78 (0.76–10.17)	1.55 (0.41–5.91)
Middle Eastern/North African	11	2 (18.2)					1.11 (0.24–5.10)	0.42 (0.06–3.10)
**Age at admission (Ref: 60–79)**	25	8 (32.0)	1.0	1.0	1.0	1.0	1.0	1.0
50–59	55	15 (27.3)	0.88 (0.37–2.08)	1.05 (0.45–2.47)	0.88 (0.37–2.08)	0.95 (0.40–2.23)	0.88 (0.37–2.08)	0.96 (0.43–2.18)
<50	70	19 (27.1)	0.86 (0.38–1.97)	0.98 (0.43–2.26)	0.86 (0.38–1.97)	0.92 (0.34–2.51)	0.86 (0.38–1.97)	0.83 (0.35–1.98)
**Sex (Ref: Female)**	91	23 (25.3)	1.0	1.0	1.0	1.0	1.0	1.0
Male	59	19 (32.2)	1.30 (0.71–2.39)	1.58 (0.88–2.83)	1.30 (0.71–2.39)	1.53 (0.83–2.83)	1.30 (0.71–2.39)	1.64 (0.89–3.02)
Charlson comorbidity index (modified)* **(Ref: <1)**	127	36 (28.3)	1.0	1.0	1.0	1.0	1.0	1.0
> = 1	23	6 (26.1)	0.87 (0.36–2.06)	0.75 (0.32–1.78)	0.87 (0.36–2.06)	0.81 (0.37–1.78)	0.87 (0.36–2.06)	0.81 (0.36–1.85)
**Diabetes (Ref: No)**	117	31 (26.5)	1.0	1.0	1.0	1.0	1.0	1.0
Yes	33	11 (33.3)	1.27 (0.64–2.53)	1.11 (0.54–2.29)	1.27 (0.64–2.53)	1.18 (0.59–2.36)	1.27 (0.64–2.53)	0.99 (0.48–2.05)
**Material and Social deprivation index (Ref: 5)**	65	22 (33.8)	1.0	1.0	1.0	1.0	1.0	1.0
1	9	1 (11.1)	0.30 (0.04–2.24)	0.36 (0.04–3.18)	0.30 (0.04–2.24)	0.28 (0.03–3.01)	0.30 (0.04–2.24)	0.34 (0.03–3.41)
2	15	7 (46.7)	1.46 (0.62–3.42)	1.66 (0.74–3.71)	1.46 (0.62–3.42)	1.58 (0.63–3.95)	1.46 (0.62–3.42)	1.66 (0.70–3.97)
3	24	5 (20.8)	0.57 (0.22–1.50)	0.56 (0.21–1.49)	0.57 (0.22–1.50)	0.48 (0.16–1.47)	0.57 (0.22–1.50)	0.58 (0.20–1.66)
4	30	6 (20.0)	0.57 (0.23–1.41)	0.55 (0.23–1.34)	0.57 (0.23–1.41)	0.48 (0.17–1.33)	0.57 (0.23–1.41)	0.54 (0.21–1.34)

*Does not include diabetes.

HR = Hazard Ratio, aHR = Adjusted HR.

### Survey results

One hundred and two HCW (68.0%) participated in the telephone survey of whom 69 (67.6%) were immigrants. Those who participated in the survey were more likely to be female (77% vs 52.9%; *p*<0.01) but were similar in all other demographic characteristics including age, immigrant status, ethnicity and region of origin compared to those who did not participate in the survey. The mean time since arrival to Canada for immigrant HCW was 23.1 years, and 83.8% (57/68) were Canadian citizens. Fifty percent (44/88) of HCW with known type of workplace worked in a RCF, and immigrant HCW were more likely to work in an RCF than non-immigrant HCW (58.6% vs. 33.3%; *p* = 0.05). One third of HCW (29.4%) worked in more than one facility, with a trend for immigrant HCW being more likely to do so than Canadian-born HCW (34.8% vs 18.2%; *p* = 0.09). A total of 86 (85.1%) HCW believed that they had been exposed at work and 85 (84.1%) of all HCW had direct contact with COVID-19 patients. Over one third of HCW (37/93) had not received any training in COVID-19 related infection prevention and control procedures and over half (49/89) did not always have access to PPE at work (Table 4).

**Table 4:** Health care worker phone survey data stratified by immigration status.

	*Overall* N(%)	*Canadian- Born* N(%)	*Immigrant* N(%)	*p-value*
**Total**	102 (100)	33 (32.4)	69 (67.6)	
**CHARACTERISTICS ON ADMISSION**				
**Age (years)**				0.41
Mean (SD)	50.1 (11.1)	48.8 (11.5)	50.7 (10.9)	
**Sex**				0.53
Male	48 (47.1)	17 (51.5)	31 (44.9)	
Female	54 (52.9)	16 (48.5)	38 (55.1)	
**Job title (N = 101)**				<0.01
Personal Support Worker	47 (46.5)	10 (30.3)	37 (54.4)	
Nurse	17 (16.8)	5 (15.2)	12 (17.6)	
Auxiliary nurse	7 (6.9)	2 (6.1)	5 (7.4)	
Physician/Medical Student	4 (4.0)	2 (6.1)	2 (2.9)	
Other Patient Contact^1^	10 (9.9)	9 (27.3)	1 (1.5)	
Other Non-Patient Contact^2^	16 (15.8)	5 (15.2)	11 (16.2)	
**Region of Birth**				
Canada	33 (32.4)	33 (100.0)		
East Asia and Pacific	9 (8.8)		9 (13.0)	
South Asia	2 (2.0)		2 (2.9)	
Middle East and North Africa	8 (7.8)		8 (11.6)	
Sub Saharan Africa	15 (14.7)		15 (21.7)	
Europe and United-States	5 (4.9)		5 (7.2)	
Latin America	6 (5.9)		6 (8.7)	
Caribbean	24 (23.5)		24 (34.8)	
**Ethnic Group**				
White	34 (33.3)	27 (81.8)	7 (10.1)	
Black	40 (39.2)	2 (6.1)	38 (55.1)	
Latino	7 (6.9)	2 (6.1)	5 (7.2)	
Middle Eastern/North African	8 (7.8)		8 (11.6)	
Asian	13 (12.7)	2 (6.1)	11 (15.9)	
**Number of years living in Canada (N = 68)**				
Mean (SD)			23.1 (13.3)	
Median (IQR)			20.0 (12.0–33.0)	
**Number of years living in Canada (categorical) (N = 68)**				
<5			7 (10.3)	
5–10			5 (7.4)	
11–20			23 (33.8)	
21–35			19 (27.9)	
36–50			12 (17.6)	
>50			2 (2.9)	
**Current Immigration Status (N = 68)**				<0.001
Citizen			57 (83.8)	
Permanent resident			6 (8.8)	
Visa: student or work			2 (2.9)	
Asylum seeker/Refugee			3 (4.4)	
**Where do you think you were exposed to COVID-19 (N = 101)**				0.51
At home (family member)	8 (7.9)	3 (9.4)	5 (7.2)	
At work	86 (85.1)	26 (81.3)	60 (87.0)	
In the community (friend, acquaintance, other)	6 (5.9)	2 (6.3)	4 (5.8)	
During travel	1 (1.0)	1 (3.1)		
**Contact with Covid-19 positive in 14 days before got sick (N = 101)**				0.38
Yes	75 (74.3)	22 (68.8)	53 (76.8)	
No	12 (11.9)	6 (18.8)	6 (8.7)	
Do not know	14 (13.9)	4 (12.5)	10 (14.5)	
**Between March and June 2020, number of workplaces**				0.09
One	72 (70.6)	27 (81.8)	45 (65.2)	
Greater than one	30 (29.4)	6 (18.2)	24 (34.8)	
**Type of Workplace (N = 88)**				0.05
Hospital Center	28 (31.8)	11 (36.7)	17 (29.3)	
Residential Care Facility	44 (50.0)	10 (33.3)	34 (58.6)	
Other	16 (18.2)	9 (30.0)	7 (12.1)	
**COVID-19 specific IPAC training (N = 93)**				0.08
Yes	54 (58.1)	14 (45.2)	40 (64.5)	
No	37 (39.8)	17 (54.8)	20 (32.3)	
Don’t know	2 (2.2)		2 (3.2)	
**Always access to PPE (N = 89)**				0.82
No	49 (55.1)	16 (53.3)	33 (55.9)	
Yes	40 (44.9)	14 (46.7)	26 (44.1)	

Abbreviations: IQR, interquartile range. NEWS2, National Early Warning Score 2. SD, standard deviation.

^1^Other Patient contact: pharmacy staff (2), social worker (2), alternative medicine (1), counsellor (1), dentist (1), neuronavigator (1), nursing student (1), respiratory therapist (1), RPA worker (1), transportation (1).

^2^Other Non–patient contact: administrative staff (9), manager (4), kitchen staff (3), housekeeping attendant (2), security (2), chemist (1), sterilization (1), recreative technician (1).

## Discussion

Our study demonstrates that HCW were overrepresented in COVID-19 associated hospitalizations in the first wave of the COVID-19 pandemic in Montréal. Many HCW were exposed while working in RCF, driven in part by inadequate access to PPE and infection control and prevention training. Immigrant HCW, many of whom were low paid essential workers in RCF were disproportionately affected. Immigrants accounted for two thirds of all hospitalized HCW and were more likely to be Black and PSW, compared to their Canadian-born colleagues. Although hospitalized HCW were younger and had fewer comorbidities, they were as likely to be admitted to the ICU compared to non-HCW, with immigrants from East Asian and the Pacific at greatest risk. HCW were less likely to die compared to non-HCW.

HCW in Quebec were among the highest-risk groups to be infected with SARS-CoV-2 in the first wave of the pandemic (March 1 to June 15, 2020). During this period, HCW were nine times more likely to test positive for SARS-CoV-2 compared to other adults aged 20–69 years, and accounted for 25% of all COVID-19 cases [29]. This risk decreased in the second wave (June 16 2020, to January 16, 2021); HCW were 3.1 more likely to test positive for SARS-CoV-2 and accounted for 12% of all cases [29]. The very high rates among HCW in the first wave of COVID-19 is likely partly explained by the fact that testing in Quebec was restricted to priority groups (including HCWs) whereas, testing became available to the general public in July, 2020. Rates in the second wave were more in line with the 3-fold increased risk among HCW reported for the US and the UK in April and May 2020 [2]. In the US and Europe, young and healthy HCW were uniformly 2–3 times more likely to be hospitalized for COVID-19 compared to the general population, thought to be due to increased SARS-CoV-2 exposure [2,7,8]. We also found that HCW were overrepresented among all COVID-19 hospitalizations, as they accounted for 14% of all hospitalizations but comprise less than 4% of the population [29].

By mid-April 2020, COVID-19 swept through public and private RCFs in Quebec with numerous outbreaks [30]. By the end of the first wave (July 2020), residents of RCFs accounted for 22% of all cases, 28% of hospitalizations, and 79% of COVID-attributed mortality [30,31]. Widespread transmission was facilitated by chronic structural deficiencies in these institutions including crowding, communal spaces, low staff-to-resident ratios, reliance on a precariously employed workforce who worked in several facilities to earn a living wage (most with no sick pay) so they could not afford to take time off work, and lack of adequate PPE [32–35]. We also found that one-third of all HCW had not received any training in COVID-19 related infection prevention and control procedures and almost half did not always have access to PPE at work [34,35].

Immigrant HCW were most vulnerable and made up two thirds of all hospitalized HCW. They were more likely to be PSW (54% vs 30%), to be Black (55% vs 6%) and there was a trend for them to be more likely to work in a RCF (59% vs. 33%) and to work in more than one health care facility (35% vs 18%) compared to Canadian-born HCW. A report of HCW infected with SARS-CoV-2 in Quebec between March 1, 2020, and May 29, 2021 similarly found that PSW and foreign born or Black HCW were 2.2, 1.3 and 2.5 times more likely to test positive for SARS-CoV-2 compared to other HCW [29]. This is similar across Canada where the majority of PSW are visible minorities; 30% of whom are Black, 30% Filipino and 15% other visible minorities [36].

In our study, HCW were as likely to be admitted to the ICU despite being younger and having fewer comorbidities, and were less likely to die compared to non-HCW with COVID-19. In adjusted analyses, after controlling for age, sex, medical comorbidities and deprivation index, HCW from East Asia and the Pacific had a 3.6-fold higher risk of ICU admission compared to their Canadian-born colleagues. Studies in the US and the UK found that migrant HCW were twice as likely as their White colleagues to be infected with COVID-19, likely due to increased exposure, and accounted for a disproportionate number of deaths [11–15]. Although migrants have worse in hospital outcomes compared to host populations, to our knowledge, there are no other studies of in-hospital outcomes among migrant HCW. Studies from Italy, Spain, the UK and our cohort including all immigrants and ethnic minorities (not just HCW), found that migrants and ethnic minorities were at increased risk of ICU admission vs. host populations after adjusting for age, sex, socio-economic status and medical comorbidities [37–40]. The consistent finding of increased risk of severe COVID-19 outcomes among hospitalized ethnic minorities and immigrants compared to host populations, despite adjustment, is unexplained and is likely due to unmeasured factors driving these inequalities such as access to and quality of care, racism, language or culture barriers [39–41]. Stress leading to increased vulnerability to infectious diseases modulated by genetic and epigenetic factors could also be a contributing factor [42,43]. Chronic stress (presumably driven by socioeconomic stressors, discrimination, or racism) among the immigrant population could therefore potentially lead to worse COVID-19 outcomes. None of these factors were measured in our study but warrant further investigation.

The first wave of the COVID-19 pandemic in Quebec highlighted several weaknesses in the health care system and provision of care in RCF that had a negative impact on the elderly population living in these settings and the HCW caring for them [34,35,40,44]. The Quebec action plan released at the end of the first wave recommended increasing the number of HCW staff in RCF and to limit HCW mobility between institutions as much as possible, while ensuring adequate number of staff [44]. A recently released Quebec Commission also made a number of suggestions to strengthen delivery of care in RCF and made similar suggestions regarding RCF staffing as well as recommending to strengthen IPAC procedures and provide access to PPE in these settings [34,35]. With the implementation of infection control measures in the community and in health care institutions and widespread access to vaccines, the situation in Quebec has significantly improved. In May 2021 the risk to HCW of testing positive for SARS-CoV2 compared to the general population was 1.8 fold, IPAC training was available in 90% of RCFs and access and adherence to PPE was improved [29]. The proportion of all cases among HCW occurring among those working in RCF decreased to 16%, however, PSW still accounted for 25% of all cases [29]. These findings underscore the fundamental importance of workplace IPAC measures and their continual refinement with advancing knowledge of COVID-19. High-risk workplaces like RCFs require design and implementation of multifaceted IPAC interventions that extend beyond PPE, quarantine and isolation to also ensuring high vaccination rates and optimizing physical infrastructure e.g. implementing adequate air handling systems and ventilation [45].

Vaccination of RCF residents and HCW has been a key strategy to control COVID-19 in these settings and a priority since the vaccine became available in December 2020 [46]. As of March 2022, 97% of all HCW in Quebec had two doses and 68% had received three doses of COVID vaccines [29,47]. These data are not stratified by occupation, ethnicity, country of birth, or immigration status and within group heterogeneity is likely. In Quebec, a recent multicentre survey of vaccine hesitancy among HCW showed that nurses and PSW were significantly less likely to be accepting of COVID-19 vaccination relative to other health care workers but these data were not stratified by immigrant status or ethnicity [48]. A study of COVID-19 vaccine hesitancy in the general population of Quebec found that 28% of people born outside of Canada were vaccine hesitant. In univariate analysis both non-white ethnicity and immigrant status were significantly associated with vaccine hesitancy [49]. Similarly, among a large cohort of HCW in the UK, ethnicity and immigrant status were associated with vaccine hesitancy [50]. Immigrants have lower vaccine uptake due to numerous barriers to vaccine access as well as vaccine hesitancy [51]. It is therefore critical to tailor vaccination strategies to the needs of migrant HCW to overcome these barriers given their high risk for SARS-CoV-2 infection.

### Limitations

We present a large study of community-acquired COVID-19 hospitalizations among four hospitals in Montréal that were at the epicenter of the first wave of the COVID-19 pandemic in Quebec and Canada. Our analyses are limited by the relatively small number of hospitalized HCW, given that they are a young and healthy population; once the data were stratified by ethnicity or country of birth, there was limited power to detect differences in the adjusted models for ICU admission among HCW. Although the survey response rate was good, the sample size of these responses was small and the presentation of the data are limited to descriptive analyses. Finally, our analyses are likely limited by residual and unmeasured confounders, as we were not able to measure factors such as BMI or access to care that may have influenced our estimates.

## Conclusions

HCW are at increased risk for COVID-19 exposure and hospitalization compared to the general population that can be prevented through strengthening infection prevention and control practices, universal access to PPE, and COVID-19 vaccination. Immigrant HCW were particularly exposed to COVID-19 infection in the first wave of the pandemic in Quebec as they were more likely to occupy low paying front facing health care jobs in RCFs where there were many outbreaks and inadequate access to IPAC training and PPE. Immigrants and ethnic minorities are also less likely to obtain COVID-19 vaccination due to barriers accessing COVID vaccination and vaccine hesitancy. Our study findings highlight the need to ensure that the most vulnerable HCW are protected from COVID-19 transmission in the workplace through strong infection control measures and high COVID-19 vaccination coverage.

## Supporting information

S1 Database (CSV)

S1 File (XLSX)
